# Complete bronchial obstruction by granuloma in a paediatric patient with translaryngeal endotracheal tube: a case report

**DOI:** 10.1186/1752-1947-8-260

**Published:** 2014-07-24

**Authors:** Nao Okuda, Emiko Nakataki, Taiga Itagaki, Mutsuo Onodera, Hideaki Imanaka, Masaji Nishimura

**Affiliations:** 1 3-18-15 Kuramoto, Tokushima 770-8503, Japan; 2 2-50-1 Kuramoto, Tokushima 770-8503, Japan

**Keywords:** Bronchial obstruction, Granuloma, Translaryngeal endotracheal tube

## Abstract

**Introduction:**

Although continuous or frequent stimuli in tracheostomized patients may cause tracheal granulomas, little is known about management of patients with translaryngeal intubation.

**Case presentation:**

A 1-month-old Japanese boy, weighing 3.5kg, was admitted to our hospital owing to cardiac failure caused by an atrial septal defect and intractable arrhythmia. To treat his unstable cardiovascular status, surgery was performed to close his atrial septal defect. After the operation, stenosis was detected by auscultation and flow limitation worsened. A bronchoscopy revealed granulomas completely obstructing his right bronchus and partially obstructing his left bronchus. Dexamethasone infusion partially reduced the mass, after which removal by yttrium aluminium garnet laser was tried. The airway obstruction was not resolved, however, because of granuloma reproliferation. Budesonide (aerosol liquid) inhalation was started, and tissue was reduced using an yttrium aluminium garnet laser and physically removed using forceps. After continued budesonide inhalation, he was successfully liberated from the ventilator.

**Conclusions:**

Life-threatening airway obstruction by granulomas developed in a translaryngeally intubated paediatric patient. The granuloma was detected after a couple of weeks of intubation. A bronchial granuloma is rare in paediatric patients. It should be suspected with evidence of bronchial obstruction. Treatment with corticosteroids and surgery using a laser maybe indicated.

## Introduction

Translaryngeal intubation causes mucosal ulcerations of the vocal cords and occasionally results in stenosis caused by a laryngeal granuloma [1]. A bronchial granuloma, however, is a less common complication. When it occurs in tracheostomized patients, it is usually attributed to continuous and frequent stimulation. Common sites where such rubbing occurs are at the tracheal orifice and around the distal tip of the tracheostomy tube. Little is known, however, about management of bronchial granulomas in patients undergoing prolonged translaryngeal intubation. We encountered a bronchial granuloma at the carina due to stimulation of translaryngeal tube in a baby.

## Case presentation

**Figure 1:**
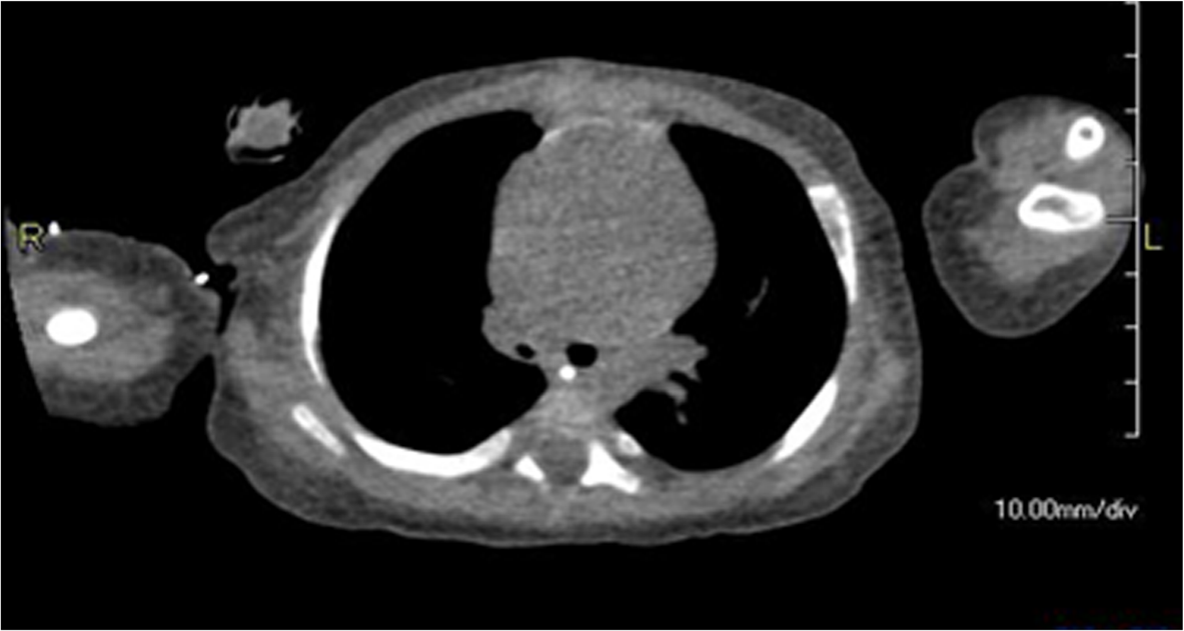
**Chest computed tomography on day 50.** Right bronchus was narrower than the left.

**Figure 2:**
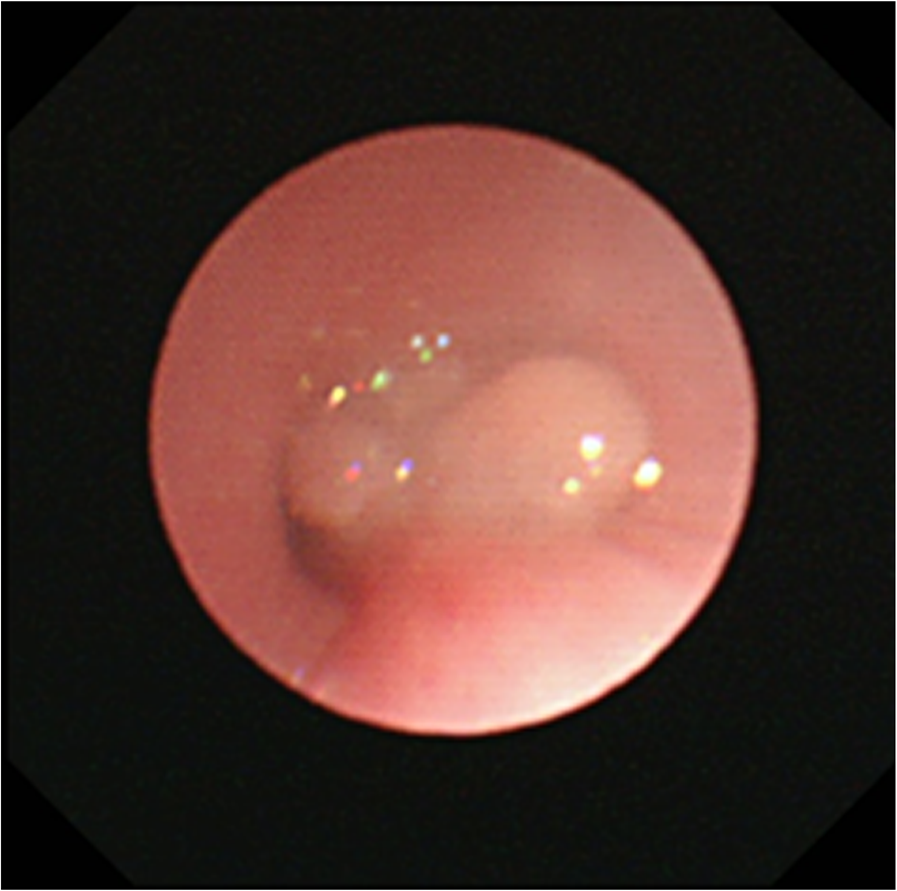
**Bronchoscopy image on day 79.** Granuloma completely obstructs the right bronchus and partially obstructs the left bronchus.

**Figure 3:**
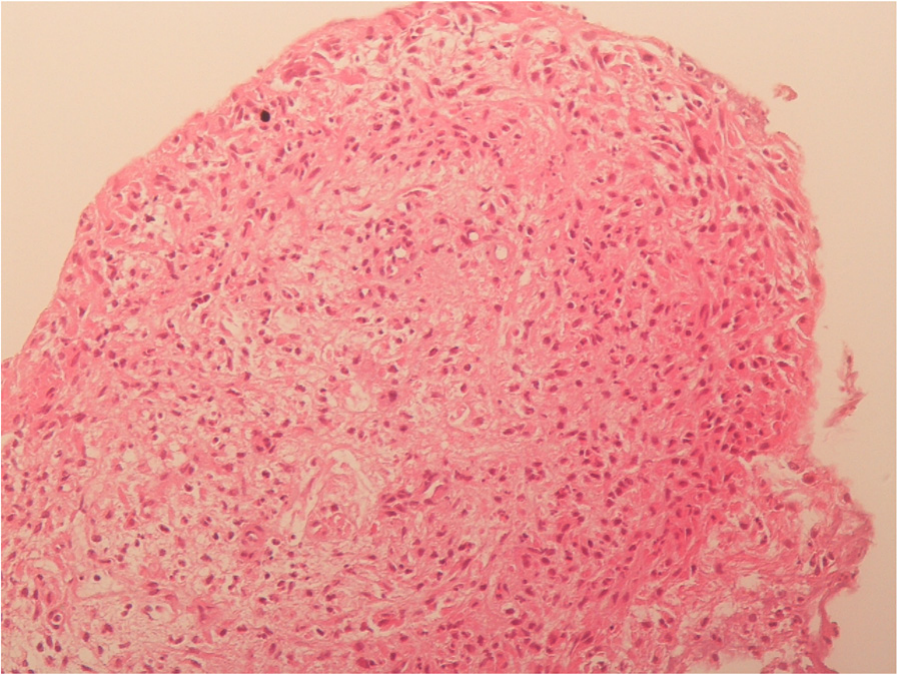
**Histological view of the granuloma.** It was diagnosed as inflammatory granuloma by a pathologist.

A 1-month-old Japanese boy, weighing 3.5kg, was admitted to our hospital with intractable arrhythmia, specifically, multifocal atrial tachycardia with heart rate greater than 180 beats per minute. The presence of an atrial septal defect (ASD) and tachyarrhythmia were causing heart failure. Because his neck was short and it was difficult to look at his epiglottis, bronchoscope-assisted transnasal intubation was performed.On day 6, perforation peritonitis was detected, and ileocecal resection and colostomy were performed. On day 20 the ASD was closed with pericardial membrane to correct the heart failure. After surgery, cardiorespiratory conditions stabilized, but evidence of stenosis was apparent from auscultation. On day 27, tracheal extubation was not possible, and re-intubation might be impossible, owing to epiglottal oedema, and the risk of upper airway obstruction after extubation. Elective tracheostomy was performed on day 48. Stenosis was still apparent during auscultation and expiratory flow limitation did not improve. A chest computed tomography (CT) scan image (Figure 1) showed narrowing of his right main bronchus and a white mass was detected by bronchoscopy. On day 79, bronchoscopy revealed an extensive granuloma in his right main bronchus just below the carina and the presence of a granuloma in the second left carina (Figures 2 and 3). His right main bronchus was occluded and even after tissue removal with forceps, granulomas proliferated again, and occlusion of the right main bronchus persisted. Consequently, from day 80 to 109 dexamethasone was administered intravenously (0.3mg/kg/day). Although the granuloma in his left bronchus disappeared, occlusion remained in his right main bronchus. Dexamethasone was gradually decreased on day 95 and replaced with prednisone 0.5mg orally on day 109. Subsequently, the granuloma increased and both bronchi were obstructed. On day 113, the granuloma was reduced with yttrium aluminium garnet (YAG) laser ablation but, a week later, bronchoscopy revealed an enlarged granuloma. Tranilast (0.05g/kg/day) was started, and on day 126, budesonide inhalation (0.5mg) was administered. On day 129, YAG laser ablation was repeated and vestiges were removed with forceps. Budesonide inhalation and tranilast were continued, and the granuloma did not reproliferate. He was weaned from mechanical ventilation, and spontaneously breathing via a tracheostomy tube; he was discharged from the intensive care unit with the tube on day 149.

## Discussion

Our patient had a life-threatening airway obstruction due to a bronchial granuloma, believed to be caused by translaryngeal intubation. After cardiac surgery, stenosis was detected by auscultation, and obstructive granuloma probably developed within 2 to 3 weeks of intubation.

A granuloma is a nodular inflammatory lesion comprising epithelioid cells and macrophages. Contact with the tip of an endotracheal tube or suction tube may stimulate the mucosal membrane to form a granuloma. Chronic stimulation or inflammation also encourages granuloma formation, which is often associated with the use of tracheostomy tubes. A granuloma has been reported in 26% to 80% of tracheostomized patients [2-4]. While noticeable formation in such patients often takes about 6 months, a laryngeal granuloma is sometimes apparent after short-term translaryngeal intubation. In this paediatric patient, tracheostomy was performed after 48 days of translaryngeal intubation. However, respiratory problems were apparent before tracheostomy, owing to aural evidence of stenosis in his right lung field after cardiac surgery. On later re-examination of the chest CT images from day 6, it was clear that his endotracheal tube must have been in contact with his carina. It is hard to be sure how long this contact persisted because, in chest X-rays, the lie of the tube tip varied with body position. Although attempts were made to carefully adjust the positioning of the endotracheal tube, maintaining deep intubation in this baby boy was challenging.

Tracheal suction was another factor. To prevent avoidable damage of epithelium around the carina, suction tubes are advised to be inserted 1cm to 2cm beyond the distal tip of the endotracheal tube. In this boy, the necessarily deeper than usual positioning of the endotracheal tube meant that the suction tube must have rubbed the endothelium at the carina and the right main bronchus more frequently than usual: granulomas developed at both main bronchi. His right main bronchus was completely occluded: we are unsure why a granuloma also developed in his left bronchus.

Although there are several treatment options for granulomas, no consensus exists about whether it is preferable to administer steroids intravenously or by inhalation, or to carry out surgical excision (with forceps or laser). It has been reported that 60% of granulomas recurred after excision and 48% of unexcised granulomas regressed spontaneously [2]. Initial intravenous administration of dexamethasone was only partially effective. After cessation of dexamethasone, a recurrent granuloma completely occluded his right bronchus. Some tissue was removed by YAG laser ablation and dexamethasone was administered intravenously, along with budesonide by inhalation. After the granuloma disappeared, the boy was liberated from mechanical ventilation. Surgical removal on this patient was indicated by the extent of the granuloma that was obstructing his bronchus.

## Conclusions

Bronchial granuloma is rare in paediatric patients. It should be suspected with evidence of bronchial obstruction. Treatment is with corticosteroids and surgery using a laser maybe indicated. To avoid granuloma formation, extra care is needed to maintain optimal endotracheal tube positioning.

## Consent

Written informed consent was obtained from the patient’s legal guardian for publication of this case report and accompanying images. A copy of the written consent is available for review by the Editor-in-Chief of this journal.

## Abbreviations

ASD: Atrial septal defect; CT: Computed tomography; YAG: Yttrium aluminium garnet.

## Competing interests

The authors declare that they have no competing interests.

## Authors’ contributions

NO and MN were major contributors in writing the case report. NO, EN, TI, MO and HI were equally responsible for data collection. MN provided critical revision of the case report. All authors read and approved the final case report.
